# Evaluation of Image Quality of Temporal Maximum Intensity Projection and Average Intensity Projection of Adaptive 4D-Spiral CT Scans: A Phantom Study

**DOI:** 10.7759/cureus.81849

**Published:** 2025-04-07

**Authors:** Hiroki Horinouchi, Toshinori Sekitani, Tatsuya Nishii, Noriyuki Negi, Keitaro Sofue, Tetsuya Fukuda, Satoru Takahashi

**Affiliations:** 1 Radiology, National Cerebral and Cardiovascular Center, Suita, JPN; 2 Radiological Technologist, Osaka College of High Technology, Osaka, JPN; 3 Radiology and Radiation Oncology, Kobe University Hospital, Kobe, JPN; 4 Radiology, Kobe University Graduate School of Medicine, Kobe, JPN; 5 Radiology, Takatsuki General Hospital, Takatsuki, JPN

**Keywords:** computed tomography angiography, computer-assisted diagnosis, four-dimensional computed tomography, image processing, tomography, whole body imaging, x-ray computed

## Abstract

Adaptive four-dimensional (4D) spiral computed tomography (CT) scans facilitate the acquisition of volume perfusion data for organs or long-range vessels; however, optimizing image quality and reducing noise while minimizing radiation doses remains challenging. Thus, image-processing techniques such as temporal maximum intensity projection (MIP) and average intensity projection (AIP) are crucial in this context. This ex vivo study aimed to compare the image noise, spatial resolution, and measurements of temporal MIP and AIP images generated from low radiation dose 4D CT scans data with those of conventional CT images using phantoms. Three phantoms were scanned with equivalent radiation doses using single helical and adaptive 10-phase 4D spiral scans using a third-generation dual-source CT scanner. Temporal MIP and AIP images of 4D CT scans were generated by summing varying numbers of phases, incorporating automatic motion correction with non-rigid registration and noise reduction algorithm. The CT values and image noise of the temporal MIP and AIP images were compared to conventional CT images. The task transfer function (TTF) was calculated using static phantoms. Vessel diameters of the phantoms for each image dataset were evaluated using motion phantoms. Temporal AIP images showed comparable CT values with those of the reference image. In contrast, the CT values of the temporal MIP images were significantly higher than those of the reference images (p<0.01). The image noise of temporal AIP images with six or more phases was equal to or lower than that of the reference images. In contrast, temporal MIP images exhibited consistently high noise levels regardless of the number of summed phases. The TTF of temporal AIP images was comparable to that of the reference CT images. However, the TTF of temporal MIP images gradually decreased as the number of summed phases increased. No significant differences were observed in vessel diameter measurements among the three groups or with varying numbers of summed phases (p>0.05). In conclusion, temporal MIP and AIP images generated from low radiation dose 4D CT scans could effectively reduce noise while preserving measurement reliability in the motion phantom, achieving performance comparable to conventional CT images.

## Introduction

Computed tomography (CT) technology has evolved to provide four-dimensional (4D) imaging through high-speed scanning, adding the dimension of time to traditional three-dimensional (3D) imaging [1]. This advancement enables the detailed visualization of temporal and spatial information for moving organs. Four-dimensional CT is particularly valuable in assessing respiratory motion and determining treatment margins for radiotherapy planning [2,3]. Additionally, 4D CT angiography is useful in evaluating cerebral flow dynamics [4,5]. However, the limited coverage area of high-speed CT scans restricts the regions available for 4D imaging, which relies on acquiring continuous multiphase data over short periods [6,7]. The introduction of 320-row area detector CTs has extended the 4D CT scans range to 160 mm [4], while third-generation dual-source CT scanners have further expanded this capability to whole-trunk 4D imaging using adaptive 4D-spiral CT [8-11]. Despite these technological advancements, the total radiation dose associated with multiphase imaging remains high. To mitigate this issue, reducing the radiation dose per phase is necessary, but this reduction often leads to increased noise and diminished image quality. To address these challenges, image processing techniques such as temporal maximum intensity projection (MIP) and temporal average intensity projection (AIP) have become essential. These techniques are instrumental in reducing noise and improving image quality while maintaining a lower radiation dose over extended acquisition times [8-15].

The temporal MIP technique evaluates enhancement over time for each voxel in 3D volume data by selecting the time point with the maximum CT values [12]. This approach can derive high-quality CT angiography from CT perfusion data, enhance arterial contrast, and reduce the required dose of contrast media. Temporal MIP images have been reported to be useful for vascular assessment [8-10]. In contrast, the temporal AIP technique calculates the average CT values over time for each voxel in the 4D CT dataset. Temporal AIP images have been reported to improve the image noise of multiphase 4D CT images [13-15]. However, both techniques require precise motion correction through non-rigid registration and noise reduction to generate diagnostic images from multiphase images with physiological variability during 4D CT scanning. The image processing techniques may potentially affect image quality and clinical measurements [8,16,17]. It is essential to comprehend the properties of each technique in order to establish the optimal 4D CT scans protocol and select a more appropriate post-processing method to achieve the target of examinations.

To date, no specific phantom studies for diagnostic imaging of the body trunk have investigated the quality of temporal MIP or AIP images. Therefore, this ex vivo study aimed to systematically compare the image noise, spatial resolution, and measurements of temporal MIP and AIP images with those of conventional CT images using phantoms. This article was previously published as a preprint on the Research Square server on October 22, 2024.

## Materials and methods

**Figure 1:**
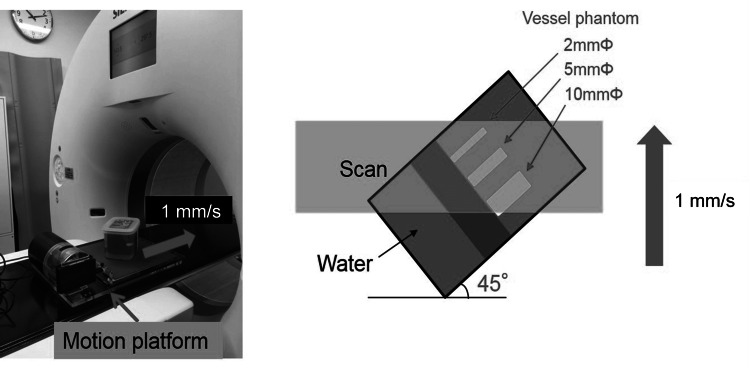
Motion platform and vessel phantoms with 2 mm, 5 mm, and 10 mm diameters. The water phantom was mounted on a motion platform that moved horizontally on a computed tomography (CT) table at 1 mm/s. Vessel phantoms with diameters of 2 mm, 5 mm, and 10 mm with a radiodensity of 300 HU were placed in the water phantom at 45° oblique to the CT scan plane.

Three ex vivo phantom studies were conducted to evaluate the performance of temporal MIP and AIP techniques. (1) CT values, image noise, and the noise power spectrum (NPS) were evaluated using a water phantom with a 20 cm diameter. (2) The task transfer function (TTF) was measured using a polyoxymethylene rod phantom with a diameter of 30 mm and 300 Hounsfield units (HU) at 120 kV in water. (3) Vessel diameter measurements in the motion phantoms on temporal MIP and AIP images were compared with those on conventional CT images acquired with a single-spiral scan. For this, a cubic water phantom containing three vessel phantoms with diameters of 2 mm, 5 mm, and 10 mm (all with a radiodensity of 300 HU at 120 kV) was mounted on a respiratory motion platform (QUASAR; Ontario, Canada: Modus QA Medical Devices) (Figure 1). The vessel phantoms were positioned at a 45° oblique angle to the CT scan plane within the water phantom. During the 10-phase 4D CT scans, the motion phantom was moved horizontally along the CT table at a constant speed of 1 mm/s, resulting in a total displacement of 25 mm.

Image acquisition and reconstruction

All CT examinations were performed using a third-generation 192-detector, dual-source CT scanner (SOMATOM Force; Forchheim, Germany: Siemens Healthineers). The phantoms underwent a single-source spiral scan for one phase as a reference and 4D scans using adaptive 4D-spiral CT scan mode (Forchheim, Germany: Siemens Healthineers) for 10 phases, with an equivalent total volume CT dose index of 10 mGy. Therefore, the volume CT dose index per phase in the 4D scans was set to 1 mGy. Detailed parameters for image acquisition and reconstruction are listed in Table 1.

**Table 1:** Acquisition and reconstruction parameters. CTDIvol: volume CT dose index ADMIRE (Forchheim, Germany: Siemens Healthineers)

Scan protocol	Single-spiral scan	Adaptive 4D-spiral scan
Tube voltage (kV)	80	80
Scan times (phases)	1	10
Radiation dose per phase (mGy)	10	1
CTDIvol (mGy)	10	10
Kernel	Bv40	Bv40
Slice thickness (mm)	1	1
Collimation	0.6×192	0.6×192
ADMIRE	3	3

All CT images were reconstructed with a 1.0 mm slice thickness using a Bv40 kernel and iterative reconstruction (ADMIRE; Forchheim, Germany: Siemens Healthineers) with a strength level of 3. The reconstructed image datasets were then transferred to the workstation (syngo.via; Forchheim, Germany: Siemens Healthineers), where temporal MIP and AIP images from the 4D CT datasets were generated using a software package (CT Dynamic Angio; Forchheim, Germany: Siemens Healthineers), which automatically incorporates motion correction with non-rigid registration and noise reduction algorithm [8,13].

Image analysis

We compared the CT values and image noise of the temporal MIP and AIP images from phases 2, 4, 6, 8, and 10 of the 4D CT scans with those of a single-spiral scan used as the reference. The CT values in HU and standard deviations (SD) were averaged across five points, namely the center of the water phantom and four points in the upper, lower, left, and right directions (appendix 1) [18]. The NPS was assessed to measure noise at each spatial frequency for the water phantom, using temporal MIP and AIP images generated by combining 2, 4, 6, 8, and 10 phases of 4D CT scans. The NPS was calculated using the radial frequency method [19,20].

Iterative reconstruction can exhibit non-linear signal characteristics that affect system resolution properties differently compared to standard filtered back projection [21]. Therefore, TTF was selected to analyze the spatial resolution of 4D CT for vessel assessment using iterative reconstruction instead of conventional modulation transfer function measurements. The in-plane resolution properties were measured in terms of the TTF using a radial edge technique on rod inserts within a water phantom [22]. The target CT value was set at 300 HU to simulate the arterial phase of the vessel.

Vessel phantoms with diameters of 2 mm, 5 mm, and 10 mm were manually measured five times by a single observer using the full width at half maximum (FWHM) method with a workstation (Ziosoft; Tokyo, Japan: Ziostation2). Measurements were performed on temporal MIP and AIP images generated by summing the 2, 4, 6, 8, and 10 phases of 4D CT scans, as well as the reference images acquired using single-spiral CT scans.

Statistical analysis

Continuous variables are expressed as mean±standard deviation. All measured values were analyzed using one-way Analysis of Variance (ANOVA) to determine differences between the parameters. Post hoc Tukey’s tests were conducted among the groups if the overall ANOVA indicated statistical significance. A p-value of less than 0.01 was considered statistically significant.

## Results

The CT values of the temporal AIP images were comparable to those of the reference CT images acquired with single-spiral scans, regardless of the number of summed phases. However, the CT values of the temporal MIP images were significantly higher than those of the reference CT images (p<0.01), and these values progressively increased with the number of summed phases (Table 2). Furthermore, the image noise in temporal AIP images with six or more phases was equal to or lower than that of the reference image, which was 9.0±0.6 HU. In contrast, temporal MIP images showed high noise levels regardless of the number of summed phases (Table 3). The NPS curves for both temporal MIP and AIP images gradually decreased as the number of summed phases increased (appendix 2).

**Table 2:** CT values measurements (in Hounsfield units). *Statistically significant difference compared with conventional CT with a single-spiral scan (10 mGy) (p<0.01). Data are presented as means±standard deviations. AIP: average intensity projection; MIP: maximum intensity projection

Conventional CT	Number of summing phases	Temporal MIP	Temporal AIP
-1.1±0.6	2	2.3±0.5*	-0.2±0.5
	4	10.0±0.8*	-0.4±0.4
	6	13.8±1.0*	-0.5±0.4
	8	16.2±1.1*	-0.5±0.5
	10	18.5±1.1*	-0.5±0.4

**Table 3:** Image noise measurements (in Hounsfield units). *Statistically significant difference compared with conventional CT with a single spiral scan (10 mGy) (p<0.01). Data are presented as means±standard deviations. AIP: average intensity projection; MIP: maximum intensity

Conventional CT	Number of summing phases	Temporal MIP	Temporal AIP
9.0±0.6	2	16.5±1.2*	16.3±1.2*
	4	13.2±1.1*	11.6±1.0*
	6	11.9±0.8*	9.3±0.7
	8	11.0±0.7*	8.1±0.5*
	10	10.8±0.6*	7.4±0.4*

**Figure 2:**
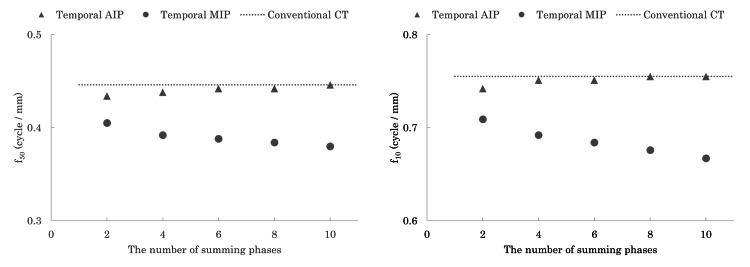
The task transfer function (TTF) of f50 and f10. The f_50_ and f_10_ from the TTF curve of temporal average intensity projection (AIP) images were similar to those of the reference conventional CT images, while those of the temporal (maximum intensity projection {MIP}) images decreased gradually with more summed phases.

**Figure 3:**
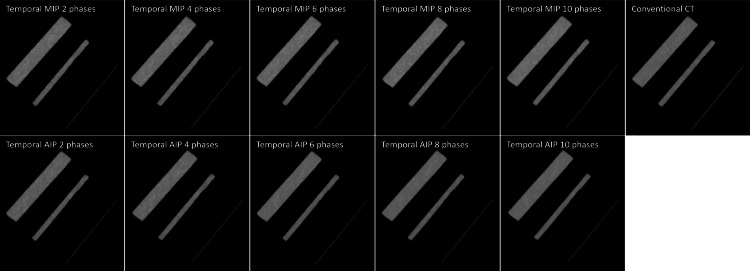
Three-dimensional maximum intensity projection images of vessel phantoms with diameters of 2 mm, 5 mm, and 10 mm with temporal MIP, temporal AIP, and conventional CT. AIP: average intensity projection; MIP: maximum intensity projection

Figure 2 illustrates the spatial frequency at which the TTF is reduced to 50% (f_50_) and 10% (f_10_) as a function of the number of summed phases. The TTF of temporal AIP images was comparable to that of the reference CT images obtained from single spiral scans. However, the TTF of the temporal MIP images decreased gradually with an increase in the number of summed phases. Table 4 shows the diameters of the vessel phantoms (2 mm, 5 mm, and 10 mm) measured using conventional CT and temporal MIP and AIP images. No significant differences in vessel diameter measurements were observed among the three groups or across the different numbers of summed phases (p>0.05). Additionally, temporal MIP and AIP images, generated from multiphase 4D CT scans of the motion phantom, showed no blurring or morphological changes when compared to conventional CT images (Figure 3).

**Table 4:** Measurements of vessel phantom diameter by full width at half maximum (FWHM). Data are presented as means±standard deviations (mm). AIP: average intensity projection; MIP: maximum intensity projection

Vessel phantom diameter	Conventional CT	The number of summing phases	Temporal MIP	Temporal AIP
10 mm	14.4±0.3	2	14.3±0.3	14.4±0.3
		4	14.3±0.3	14.4±0.3
		6	14.4±0.2	14.2±0.2
		8	14.5±0.2	14.3±0.2
		10	14.5±0.2	14.3±0.2
5 mm	5.9±0.2	2	6.1±0.1	6.0±0.1
		4	6.1±0.2	6.1±0.2
		6	6.0±0.1	6.0±0.1
		8	6.0±0.1	6.0±0.1
		10	6.0±0.1	6.0±0.1
2 mm	2.2±0.3	2	2.1±0.2	2.1±0.2
		4	2.1±0.2	2.1±0.2
		6	2.1±0.2	2.1±0.2
		8	2.2±0.2	2.2±0.2
		10	2.2±0.2	2.2±0.2

## Discussion

In this study, both temporal MIP and AIP techniques demonstrated a reduction in noise as more phases were summed. The image noise in temporal AIP images with more than six phases was equal to or lower than that of conventional CT images with a comparable spatial resolution. In contrast, temporal MIP images exhibited significantly higher CT values and substantially more severe image noise compared to conventional CT images. Despite these variations, no significant differences were observed in the diameter measurements of temporal MIP and AIP images when compared to conventional CT images, even when summing multiphase 4D CT images of the motion phantom.

Temporal AIP images generated using six phases (60%) of the 4D CT image data exhibited noise levels comparable to those of conventional CT images. This result is noteworthy because each phase of the 4D CT images, when taken at a low radiation dose, typically results in higher noise levels. We attribute this outcome to the denoising algorithm applied and the cumulative denoising effect of additive averaging of the images. Furthermore, the AIP images showed consistent CT values, making AIP a viable option in clinical settings where preserving CT values and achieving low-noise images are essential. Temporal AIP technique has been reported to improve myocardial delayed enhancement and extracellular volume CT images through the reduction of image noise with minimal effect on CT values [13-15].

In contrast, the CT values in temporal MIP images gradually increased with the number of summing phases, as temporal MIP captures the maximum CT values over time for each voxel. This study showed significantly higher CT values in temporal MIP images than those of conventional CT images. However, the unique characteristics of temporal MIP make it particularly useful for vascular assessment. Temporal MIP technique can enhance intravascular CT values to clinically appropriate levels, even with 4D CT images obtained with reduced contrast media doses. Temporal MIP images have already been reported to generate high-quality CT angiography, increase arterial contrast enhancement, and reduce both contrast media dose and image noise [8-10].

When summing techniques such as temporal MIP and AIP were applied to multiphase 4D CT images using the motion phantom with linear movement, no changes in the vessel diameters were observed. Temporal MIP images had an insignificant effect on vessel phantom measurements and did not significantly deteriorate spatial resolution, as assessed using TTF. These minimal changes in spatial resolution are considered acceptable, especially within the body trunk region, including organs with less movement. However, it is important to note that physiological variability in organs and vessels includes not only movement but also morphological changes. Temporal AIP and MIP images can effectively measure smaller lung masses targeted in radiotherapy; however, their application may be limited in cases involving significant deformations or distortions, or images with motion artifacts owing to large movements during multiphase imaging [16]. Consequently, temporal MIP and AIP images generated from 4D CT scans are more suitable for regions with less physiological variability, such as the brain and aorta [8-10,12]. In clinical practice, substantial knowledge of the advantages and disadvantages of each technique can maximize the potential of 4D CT scanning to achieve the target of the examinations.

This study had several limitations. First, complex movements and morphological changes in the phantoms were not evaluated. Complex physiological movements such as cardiac, respiratory, and peristaltic motions can cause significant morphological changes over longer acquisition times across multiple phases. These changes may lead to a deterioration in image noise, spatial resolution, and measurement accuracy in temporal MIP and AIP images. Second, this study did not consider the effect of contrast bolus flow on image quality. For instance, in the case of the aorta, MIP demonstrates the most contrast-enhanced CT values when a time series is captured before, during, and after the arrival of the contrast bolus. In contrast, AIP averages the CT values in the aorta, resulting in lower CT values than those observed with MIP. These effects on image quality in clinical practice should be addressed in future studies.

## Conclusions

In conclusion, temporal MIP and AIP images generated from low radiation dose 4D CT scans could effectively reduce noise while preserving measurement reliability in a motion phantom, achieving performance comparable to conventional CT images. These techniques contribute to minimizing radiation exposure during prolonged acquisitions of 4D CT. However, it is essential to note that temporal MIP images yield the highest CT values, whereas temporal AIP images represent the time-averaged CT values for each voxel in 4D CT images. This distinction is critical when interpreting CT values in the generated images. Additionally, this study’s phantoms did not account for complex physiological movements or morphological changes, which should be considered when applying these findings to clinical practice. A thorough understanding of the strengths and limitations of each technique is essential for optimizing 4D CT scanning to achieve specific diagnostic goals.

## Figures and Tables

**Figure 4:**
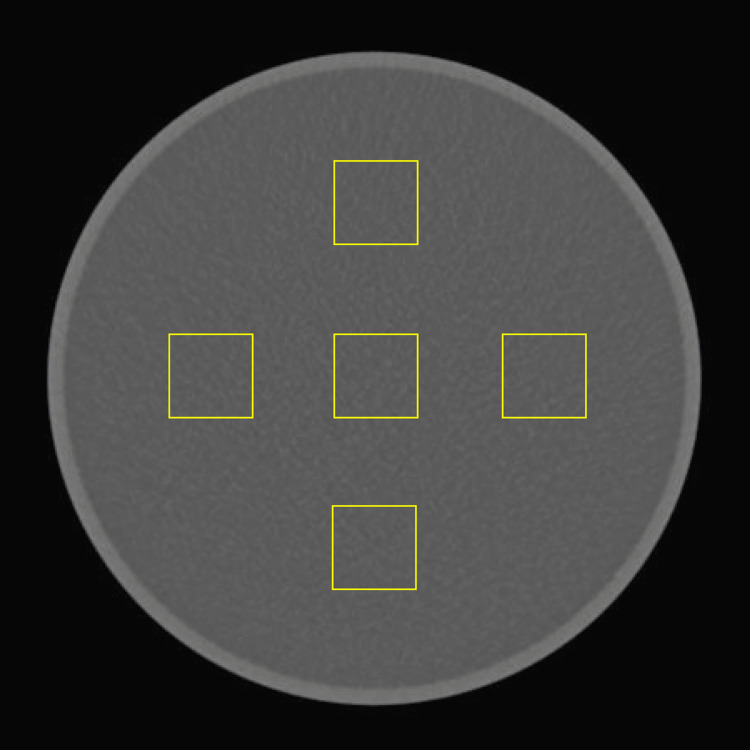
CT values measurements for regions of interest (ROI) in the water phantom. The CT values were averaged across five ROIs of 50×50 pixels placed at the center and the upper, lower, left, and right directions of the 20 cm diameter water phantom. The CT values in Hounsfield units and standard deviations of the temporal maximum intensity projection and average intensity projection images from phases 2, 4, 6, 8, and 10 of the 4D-CT scans were compared with the reference.

**Figure 5:**
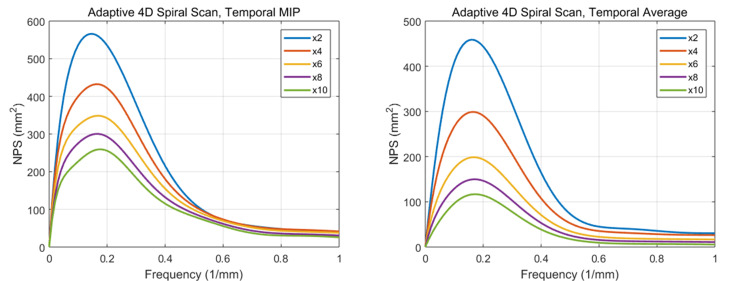
Noise power spectrum (NPS) of temporal maximum intensity projection and average intensity projection summing varying phases (2-10 phases).

## Appendices

Appendix 1

Appendix 2
